# The chemokine receptor CXCR7 interacts with EGFR to promote breast cancer cell proliferation

**DOI:** 10.1186/1476-4598-13-198

**Published:** 2014-08-28

**Authors:** Nicole Salazar, Daniel Muñoz, Georgios Kallifatidis, Rajendra K Singh, Mercè Jordà, Bal L Lokeshwar

**Affiliations:** Sheila and David Fuente Graduate Program in Cancer Biology, University of Miami Miller School of Medicine, Miami, FL USA; Research Service and GRECC, Miami VHA Medical Center, Miami, FL USA; Department of Urology (M-800), University of Miami Miller School of Medicine, PO Box 016960, Miami, FL 33101 USA; Department of Pathology, University of Miami Miller School of Medicine, Miami, FL USA

**Keywords:** Chemokine receptor, CXCR7, EGFR, Heterodimerization, β-arrestin2, Breast cancer cell proliferation

## Abstract

**Background:**

Recent advances have revealed a significant contribution of chemokines and their receptors in tumor growth, survival after chemotherapy, and organ-specific metastasis. The CXC chemokine receptor-7 (CXCR7) is the latest chemokine receptor implicated in cancer. Although over expressed in breast cancer cell lines and tumor tissues, its mechanism of action in breast cancer (BrCa) growth and metastasis is unclear. Studies in other cancers have implicated CXCR7 in cell proliferation, anti-apoptotic activity and cell-cell adhesion. The present study was initiated to examine the pattern of CXCR7 expression and its role in regulation of growth signaling in breast cancer.

**Methods:**

The contribution of CXCR7 in BrCa cell proliferation was investigated in representative cell lines using real time quantitative PCR (q-PCR), proliferation assays, immunohistochemistry and immunoblotting. Phenotypic changes were examined after CXCR7 specific cDNA and siRNA transfection and expression levels were monitored by q-PCR. Further, the association of CXCR7 with epidermal growth factor receptor (EGFR) and modulation of its activity were investigated by western blotting, immunofluorescence, and in-situ proximity ligation assays in human BrCa cells and tissues.

**Results:**

CXCR7 was expressed in both, estrogen receptor (ER) positive and negative BrCa cell lines. CXCR7 was also expressed unevenly in normal breast tissues and to a much higher extent in ER + cancer tissues. Depletion of CXCR7 in MCF7 BrCa cells by RNA interference decreased proliferation and caused cell cycle arrest. Further, proximity ligation assay (PLA) revealed colocalization of CXCR7 with EGFR in cancer tissues and cancer cell lines. CXCR7 depletion reduced levels of phospho-EGFR at Tyrosine1110 after EGF-stimulation and also reduced phosphorylation of ERK1/2, indicating a potentially direct impact on mitogenic signaling in MCF7 cells. Using siRNA to knockdown β-arrestin2 in cells with EGFR over expression we were able to nearly deplete the CXCR7-EGFR colocalization events, suggesting that β-arrestin2 acts as a scaffold to enhance CXCR7 dependent activation of EGFR after EGF stimulation.

**Conclusions:**

These results demonstrate coupling of CXCR7 with EGFR to regulate proliferation of BrCa cells and suggest an important ligand-independent role of CXCR7 in BrCa growth. Thus, the CXCR7-EGFR axis is a promising target for breast cancer therapy.

**Electronic supplementary material:**

The online version of this article (doi:10.1186/1476-4598-13-198) contains supplementary material, which is available to authorized users.

## Background

CXCR7 is a seven-transmembrane receptor that binds chemokines CXCL11/ITAC, CXCL12/SDF-1a, and macrophage migratory inhibitory factor (MIF) [1, 2]. CXCR7 can form homo-dimers and hetero-dimerize with CXCR4 [3, 4]. CXCR7 binding to the chemokine SDF-1a induces a gradient shift critical for correct development and primordial germ cell migration which led to its reputation as a scavenger receptor [5]. CXCR7 is considered an atypical chemokine receptor for several important reasons. It has a modified amino acid motif (DRYLAIV) at the second intracellular loop, which prevents it from coupling to G-proteins and inducing intracellular Ca^2+^ mobilization [6, 7]. Therefore, CXCR7 does not signal through the classical G-protein coupled receptor (GPCR) mechanism of secondary messengers. Instead it has been shown to interact with β-arrestin2 (β-AR2) as an accessory protein in a ligand dependent manner [8]. β-arrestins normally dock onto the phosphorylated cytoplasmic tail of an activated receptor, thus preventing further activation or downstream signaling, because they block the G proteins from docking onto the receptor. β-arrestins may play other roles by acting as scaffolds. The arrestin scaffolds may serve as adapter molecules to assemble multi-protein complexes ultimately leading to receptor internalization, recycling back to the plasma membrane, and downstream signaling events, including ERK1/2 (extracellular signal-regulated kinases) activation [9–11]. Arrestins may also shuttle between the cell nucleus and cytoplasm [12]. This process is not fully elucidated for CXCR7.

The behavior of CXCR7 is tissue and context dependent. It plays an important role in development and potentially in the progression of cancer to the metastatic stage. CXCR7 has been found to be expressed in human breast, lung, and prostate cancers in a stage-and grade specific pattern [13, 14]. Increased expression of CXCR7 is attributed to IL-8 (Interleukin-8), inactivation of HIC1 (hypermethylated in cancer-1), activation of HIF-1α (hypoxia-inducible factor-1), and activation of NF-kB (nuclear factor kappa B) [15–20].

The regulation of BrCa growth by chemokine receptor and growth factor receptor interaction is a relatively nascent area of research. The innate heterogeneity of BrCa adds to its complexity, therefore proliferation mechanisms may be altered during cancer progression. CXCR7 or other relevant proteins may contribute to proliferation and may be significant targets for improved cancer therapy.

In this study we show that CXCR7 is an important modulator of cell proliferation and cell cycle progression of CXCR7-expressing BrCa cells. We demonstrate that in BrCa, CXCR7 co-localizes with EGFR and CD31. We show that down-regulation of CXCR7 affects the phosphorylation status of EGFR and partially decreases the phosphorylation of key mediators of the MAPK (mitogen-activated protein kinases) cascade leading to cell cycle arrest. We also show that CXCR7 interacts with EGFR in BrCa tissue and that in some BrCa cells this relationship may significantly contribute to BrCa proliferation in a ligand independent fashion in concert with β-AR2.

## Results

### CXCR7 is expressed across breast cancer cell lines

**Figure 1:**
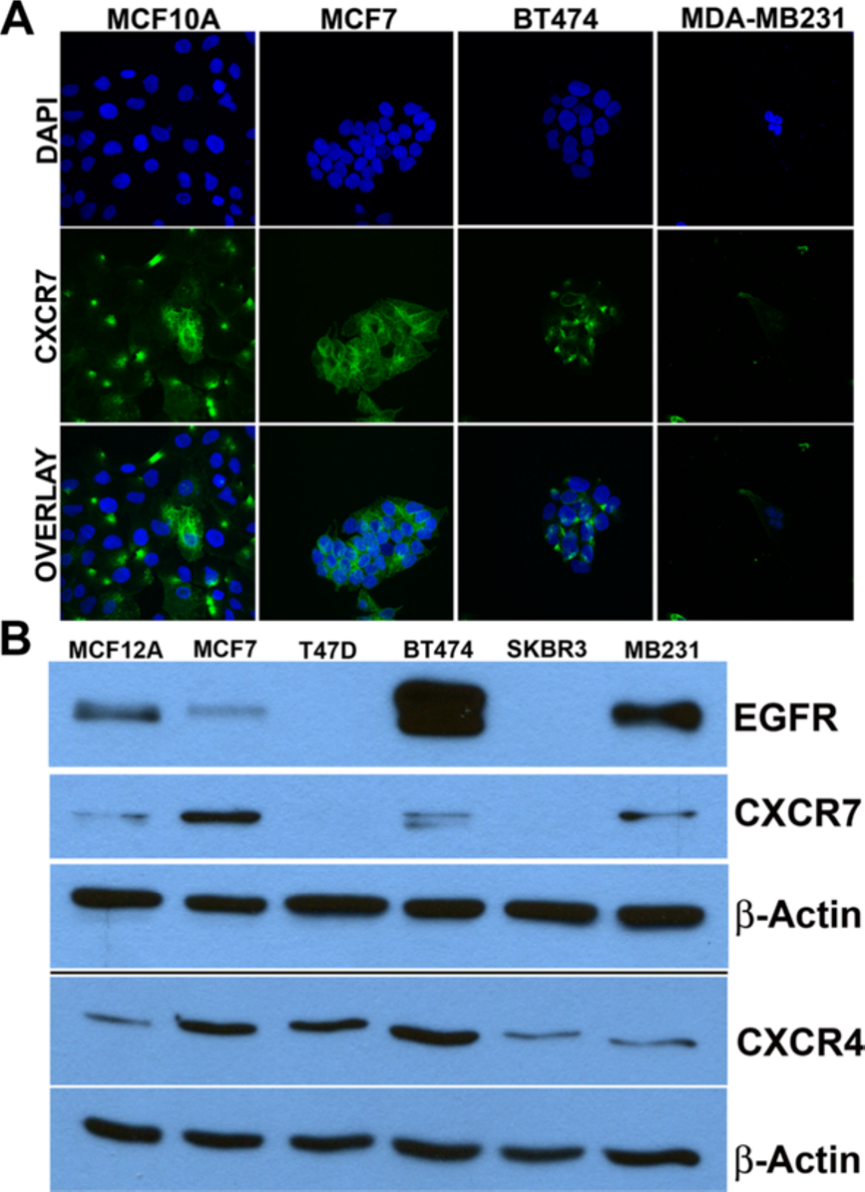
**Validation of CXCR7 expression in BrCa cell line lines through immunofluorescence and western blot analysis.** Panel **A**. CXCR7 protein expression (green fluorescence) was detected through immunofluorescence across some of the most commonly used BrCa cell lines. Panel **B**. In parallel experiments, cells were seeded in cell culture plates and 24 h later cells were lysed, proteins were collected and quantified and CXCR7, CXCR4, and EGFR expression was analyzed by Western blot analysis as described in Material and Methods. Actin served as a loading control.

We performed q-PCR to evaluate the expression level of CXCR7 in breast cancer cell lines. Table 1 shows CXCR7 mRNA expression relative to the housekeeping gene PPIA. We also included CXCR4 mRNA expression for comparison of levels of CXCR4 vs CXCR7. The cell lines are categorized as previously described by Neve et al. [21]. CXCR7 is considered over-expressed (>2 fold difference) across established BrCa cell lines when compared to normal epithelial breast cell line MCF12A or MCF10A. CXCR7 was expressed in both estrogen receptor positive (ER+) (MCF7, T47D, BT474 and HCC 202) and estrogen receptor negative (ER-) (HCC1954, HCC1569) cells. However, CXCR7 expression was highest in the ER + luminal cells. CXCR7 was expressed by HER2 over-expressing BT474 cells, however, not expressed in HER2 over-expressing SkBr3 cells. Moreover, CXCR7 was expressed at a low level in the aggressive post-EMT cell line MDA-MB231. Confocal microscopy and Western blot analysis were performed to validate expression of CXCR7 in breast cancer cells at the protein level in the most common cell lines (Figure 1). The breast cancer cell lines MCF7 and BT474 showed the highest CXCR7 expression at both mRNA and protein level compared to the normal epithelial cell line MCF12A. No CXCR7 protein expression was observed in T47D and SKBR3 cells. EGFR protein expression was also assessed to compare to CXCR7 protein expression. No EGFR was detected in T47D and SKBR3 cells and CXCR4 was expressed across all BrCa lines tested.

**Table 1:** **CXCR7 and CXCR4 mRNA level relative to PPIA (FD)**

Cell line	ER	PR	HER2	Tumor type	Classification	CXCR7	CXCR4
MCF10A*	-	-	-	F	Basal B	0.07	0.03
MCF12A*	-	-	-	F	Basal B	0.01	0.47
**MCF7**	+	+	-	IDC	Luminal	0.53	0.03
**T47D**	+	+	-	IDC	Luminal	0.04	0.06
**BT474**	+	+	+	IDC	Luminal	0.94	0.20
**SKBR3**	-	-	+	AC	Luminal	0.00	0.19
**MDAMB231**	-	-	-	AC	Basal B	0.04	0.19
**HCC202**	-	-	+	Duc Ca	Luminal	0.15	0.07
**HCC 1569**	-	-	+	MC	Basal	0.02	0.03
**HCC 1954**	-	-	+	Duc Ca	Basal A	0.03	0.03

CXCR7 and CXCR4 mRNA expression relative to PPIA expression was analyzed by q-PCR. The cell line classification based on estrogen receptor (ER), progesterone receptor (PR) and human epidermal growth factor receptor 2 (HER2) status was also included in this table as previously described in the literature [21].

*immortalized normal epithelial cells.

F = Fibrocystic disease.

IDC = invasive ductal carcinoma.

AC = adenocarcinoma.

MC = metastatic carcinoma.

Duc Ca = Ductal carcinoma.

### CXCR7 expression affects proliferation of breast cancer cells in a ligand and co-receptor independent manner

**Figure 2:**
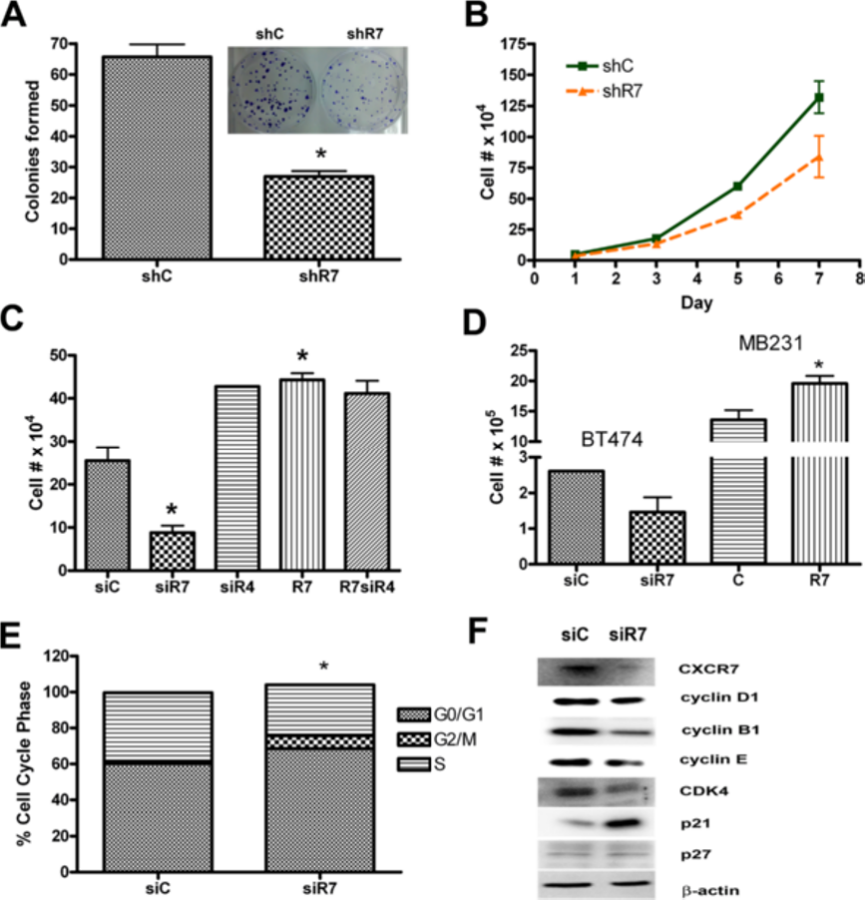
**CXCR7 depletion in MCF7 cells affects breast cancer cell proliferation. A**. The clonogenic potential of CXCR7 depleted MCF7 cells was analyzed in colony forming assays. The insert shows colony formation in CXCR7 depleted (stably shCXCR7 (shr7) transfected cells, right well) compared to vector control transfected cells (shC) (left well). **B**. Cell proliferation of MCF7 cells with stable CXCR7 depletion (shCXCR7 clone, (shR7)) compared to control cells (shC). **C**. Effect of CXCR7- (siR7) and CXCR4- (siR4) down regulation and CXCR7 (R7) over expression on proliferation of MCF7 cells. **D**. Proliferation of CXCR7 depleted (siR7) BT474 cells and CXCR7 over expressing (R7) MB231 cells compared to corresponding controls (siC = control siRNA, C = empty plasmid control). **E**. Cell cycle arrest of cells with down regulated CXCR7. **F**. Analysis of relevant cell cycle markers following siRNA mediated down regulation of CXCR7. Asterisks indicate statistical significance, P < 0.05.

Since CXCR7 was abundantly expressed in several ER + BrCa cells, we investigated the role of CXCR7 in breast cancer cell proliferation. We down regulated CXCR7 expression using RNA interference. Transient transfection of CXCR7 siRNA resulted in an 80 ± 5% decrease of mRNA levels as quantified by q-PCR, i.e. the Ct difference averaged 2.18 for MCF7 siRNA control (Ct 19.95) vs MCF7 siCXCR7 (Ct 22.13) in MCF7 cells. Protein expression was quantified using densitometry software. Figure 2F shows the siCXCR7 lane with an 86% decrease in CXCR7 protein expression compared to the siRNA control lane. The stable shRNA CXCR7 levels averaged approximately 50% less CXCR7 protein expression than the shRNA vector control. CXCR7 down regulation resulted in a 67% decrease of clonogenic growth (Figure 2B), and decreased cell proliferation in stably transfected cell lines (Figure 2A-B). To assess whether CXCR7 cooperates with its known co-receptor CXCR4 to mediate proliferation we used siRNA to knockdown CXCR4 expression. We observed significant decreased proliferation only after CXCR7 knockdown and increased proliferation with CXCR7 over expression (Figure 2C). Down regulation of CXCR4 had no significant decrease on proliferation compared to control. Down regulation of CXCR7 in BT474 cells also resulted in decreased cell proliferation. We also over expressed CXCR7 in a cell line with low endogenous CXCR7, MDA-MB231, where over expression of CXCR7 resulted in increased cell proliferation (Figure 2D).

### CXCR7 regulates the cell cycle machinery

To elucidate the inhibition of proliferation following CXCR7 down-regulation, we performed cell cycle phase fractionation analysis of propidium iodide labeled cells using flow cytometry. The results showed significant increase in the G0/G1 fraction (33%) and a significant decrease in the S-phase fraction in siCXCR7 MCF7 cells compared to control siRNA-transfected cells (Figure 2E). Importantly, CXCR7 down regulation led to marked changes in key proteins involved in regulating the cell cycle. CXCR7 depletion in MCF7 cells affected the levels of proteins regulating the S-phase transition and proliferation. The proliferative markers Cyclin B1 and Cdk4 decreased, while S-phase inhibitory protein, p21 was dramatically increased in CXCR7 depleted cells (Figure 2F). To test whether CXCR7 depletion had an effect on apoptosis, we used cell surface annexin-V labeling. Results of annexin-V binding assays on CXCR7 depleted cultures at 48 h and 72 h following siRNA transfection showed that down regulation of CXCR7 (CXCR7siRNA) did not induce apoptosis compared to cells transfected with control siRNA (Data not shown).

### Ligand independent effect of CXCR7

**Figure 3:**
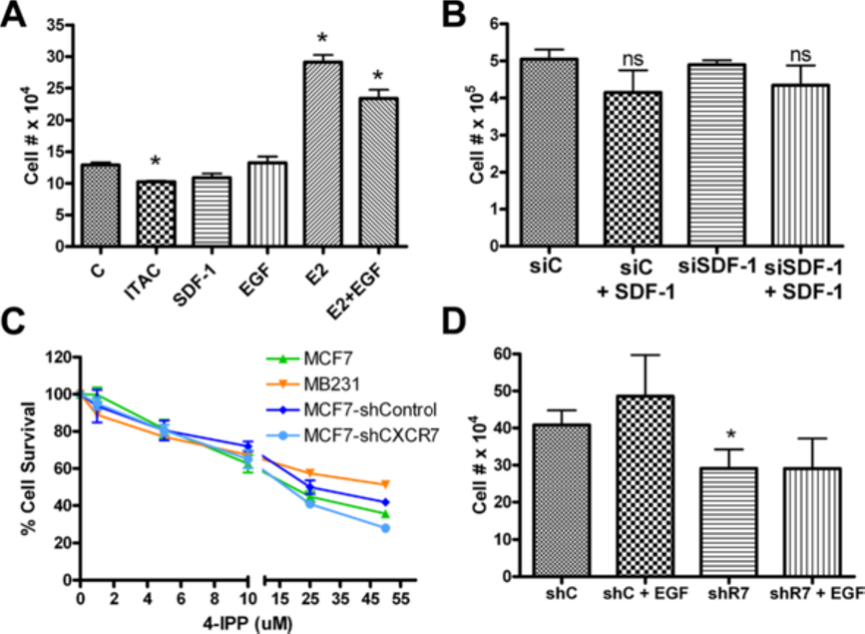
**CXCR7 ligand independent effect. A**. MCF7 cells were stimulated with CXCR7 ligands CXCL11 (ITAC), CXCL12 (SDF-1a) and proliferation was evaluated. A significant increase in growth was observed in cultures treated with positive controls, estrogen (E2) or combination of estrogen with epidermal growth factor (E2 + EGF). **B**. Effect of endogenous and external added SDF-1a on MCF7 cell proliferation. Rescue of potential SDF-1a mediated autocrine activation of CXCR7 using siRNA to down regulate endogenous SDF1a (siSDF-1) compared to control siRNA (siC) and addition of external SDF-1a to media (+SDF-1); ns: no statistical difference. **C**. Percent survival of cells based on quantification of optical density measurements of cells treated 4-IPP, a specific MIF inhibitor for 48 hrs. MTT assay was used for viability estimation. **D**. Proliferation experiments comparing the stable line MCF7 vector shRNA control (shC) and shCXCR7 (shR7) treated with EGF (10 ng/mL) for 72 hrs. Data presented from one representative experiment with triplicate determination of cell numbers.

We hypothesized that CXCR7 induces proliferation in CXCR7 expressing cells due to ligand-mediated activation of the mitogenic pathway. To test this hypothesis, MCF7 cell cultures were starved of growth factors for 24 h and then stimulated with ITAC, SDF-1a, or EGF. Proliferation was evaluated 48 h later. As shown in Figure 3A, no significant increase in cell numbers were observed between untreated and ITAC or SDF-1a treated cultures. ITAC stimulation only marginally decreased proliferation. However, a significant increase in growth was observed in cultures treated with positive controls, estrogen (E2) or combination of estrogen with epidermal growth factor (E2 + EGF). It has been reported that MCF7 cells produce the CXCR7 ligand SDF-1a in an estrogen-dependent manner [22]. To verify whether endogenous SDF-1a induces CXCR7 mediated proliferation, we transfected the cells with siRNA against SDF-1a, incubated them in presence or absence of SDF-1a and then assessed proliferation. Down regulation of SDF-1a caused no significant changes in cell proliferation (Figure 3B), neither did supplementation with SDF-1a cause increased growth. To determine if the non-canonical ligand MIF had a proliferation effect mediated through CXCR7, we added a specific inhibitor of MIF, 4-IPP, to breast cancer cell lines with distinct CXCR7 levels. As shown in Figure 3C, we found that 4-IPP inhibited growth of all tested cell lines. However, we found no association of 4-IPP cytotoxicity with CXCR7 expression. For example, 4-IPP was toxic to both MCF7 cells and CXCR7-depleted (shCXCR7) MCF7 cells. Similarly, it was cytotoxic to MB231 which express low levels of CXCR7 (Figure 3C).We investigated the relative proliferation levels of MCF7 cells with stable expression of vector shRNA (shC, control) and shCXCR7 (shR7) in the presence of EGF (10 ng/mL) for 72 hrs by counting the cells at the end of incubation. In the control cells there was a marginal increase in proliferation in EGF stimulated cells vs non stimulated cells, as seen in Figure 3A. In cells with CXCR7 depletion, shR7, there was no apparent or significant increase in EGF stimulated vs non stimulated shCXCR7 cells (Figure 3D).

### CXCR7 co-localizes with CD-31 and EGFR in the human breast cancer cell line MCF7 and breast cancer tissue

**Figure 4:**
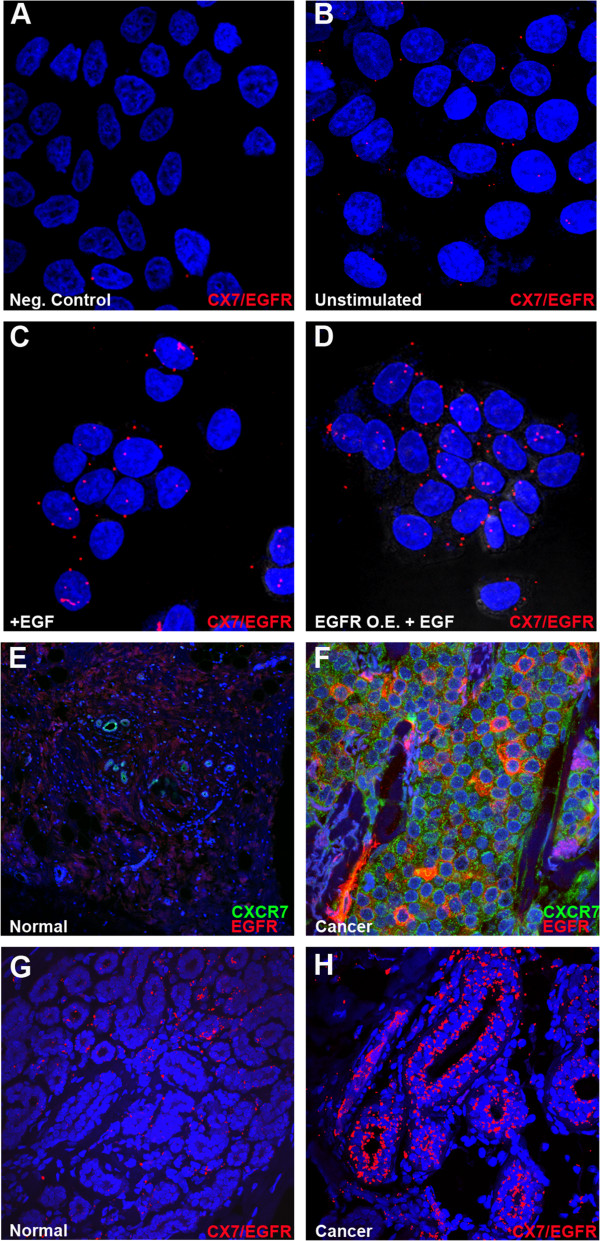
**CXCR7 co-localizes with EGFR. A-D**. After fixation of MCF7 cells, PLA (in situ co-immunoprecipitation) was performed with specific CXCR7 [GTX100027, (1:500), GeneTex, Irvine, CA] and EGFR antibodies [E2760, (1:500), Sigma Aldrich, St. Louis, MO] to visualize heterodimerization of CXCR7 with EGFR. Colocalization is shown as a red fluorescent PLA signal (CX7/EGFR), and nuclei were counterstained with DAPI (blue) (630x magnification). As the PLA technique requires two specific antibodies to give a red fluorescent signal we used a single primary antibody as a negative signal control. **A**. Negative controls experiment using EGFR antibody alone. **B**. Basal level of CXCR7-EGFR colocalization in non-stimulated MCF7 cells. **C**. CXCR7-EGFR colocalization in MCF7 cells stimulated for 2 min with EGF (10 ng/mL). **D**. CXCR7-EGFR colocalization in EGFR over expressing (O.E.) MCF7 cells following 2 min EGF (10 ng/mL) stimulation. **E**. Immunofluorescence of normal human breast tissue for detection of CXCR7 (green fluorescence) and EGFR (red fluorescence) (400× magnification). **F**. Immunofluorescence of ER + breast cancer tissue stained as explained in **E. G**. PLA of normal human breast tissue using CXCR7 and EGFR antibodies to visualize colocalization of CXCR7 with EGFR (CX7/EGFR), nuclei were counterstained with DAPI; 400× magnification. **H**. PLA of ER + human breast tumor tissue stained as explained in **G**.

Since CXCR7 affects proliferation in BrCa cells independently of its ligands and co-receptor CXCR4, we aimed to evaluate which proteins associate with CXCR7 and participate in CXCR7 mediated proliferation. We first evaluated co-localization/interaction of CXCR7 and EGFR in MCF7 cells. We performed a specific immunofluorescence approach, an in-cell co-immunoprecipitation assay, also known as a proximity ligation assay (PLA). Figure 4 demonstrates co-localization of CXCR7 with EGFR in MCF7 cells. Figure 4B shows co-localization of CXCR7 and EGFR in the absence of 10 ng/ml EGF stimulation. However, the CXCR7-EGFR co-localization was dramatically increased upon stimulation with EGF for 2–5 minutes (Figure 4C). Furthermore, we used an EGFR plasmid to minimally over express EGFR as MCF7 cells express a relatively low level of EGFR and observed a further increase of CXCR7-EGFR co-localization compared to negative controls (Figure 4D). Using immunofluorescence (IF), we also found that CXCR7 and EGFR co-localized strongly in breast cancer tissue compared to normal breast tissue (Figure 4E-F). We went futher to analyze for CXCR7-EGFR co-localization events in human breast tissue using the PLA. We confirmed an increased CXCR7-EGFR interaction in human ER + breast cancer tissues compared to normal breast tissues (Figure 4G-H). As CXCR7 is known to be expressed in endothelial cells and tumor associated vasculature of bladder and prostate cancers, we verified the expression of CXCR7 in the context of the endothelial cell marker CD31 in BrCa tissues. We used IF to evaluate expression and co-localization of endothelial cell marker CD31 with CXCR7 in human breast tissue. Additional file 1: Figure S1 shows that CXCR7 co-localizes with CD31, which is consistent with its reported expression in endothelium. Moreover, in order to assess whether CXCR7 expression is aberrant in the breast tumor microenvironment vs. normal tissue, we used the Oncomine data base to query. Our analysis using the Ma Breast 4 database [23] indicates that CXCR7 expression is higher in breast cancer tissue stroma compared to normal breast tissue stroma.

### Down regulation of CXCR7 decreases levels of p-ERK and p-EGFR in MCF7 cells

**Figure 5:**
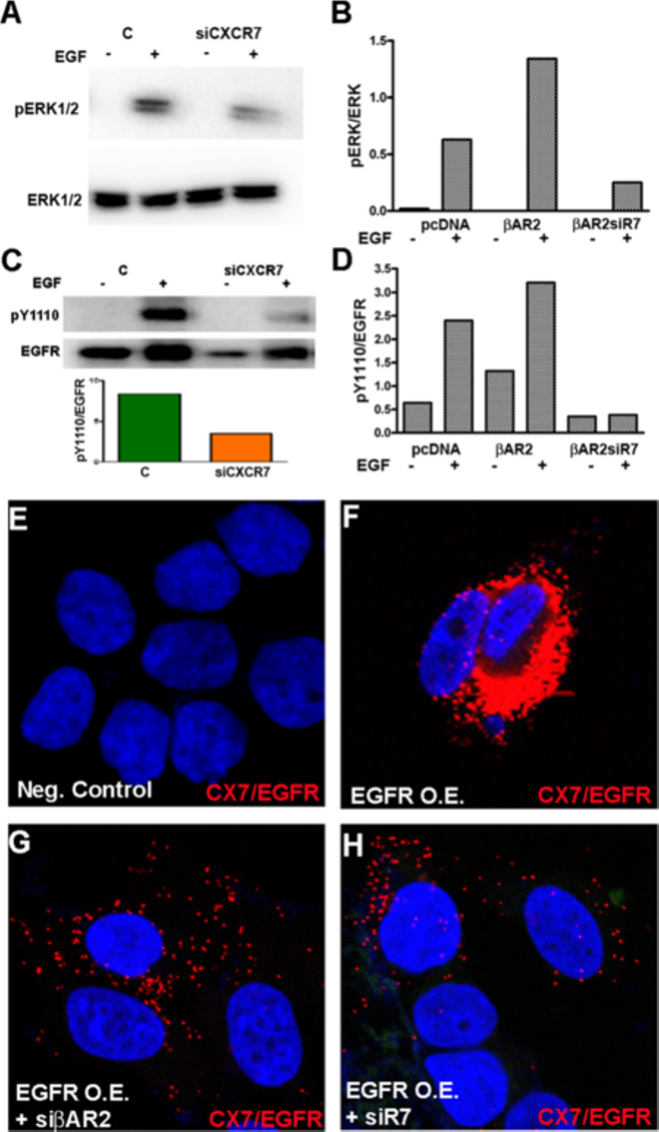
**β-arrestin2 plays a role in CXCR7 mediated phosphorylation of EGFR and ERK. A**. Levels of pERK1/2 and total ERK in MCF7 cells transfected with siCXR7 or control siRNA (C). ERK levels were determined by Western blotting of cell lysates prepared from serum-starved cells stimulated with EGF (10 ng/mL) for 5 minutes. **B**. The ratio of p-ERK1/2 over total ERK1/2 in western blot was quantified from analysis of MCF7 cell lysates that were transfected with control plasmid (cDNA), β-arrestin2 (βAR2) or β-arrestin2 in combination with siCXR7 (βAR2siR7) and stimulated with EGF (+). **C**. CXCR7 down regulation via siRNA decreased activation of EGFR at Tyrosine 1110 in MCF7 cells. **D**. The ratio of p-EGFR_Y1110_ over total EGFR was quantified in cells transfected with control plasmid (cDNA), β-arrestin2 (βAR2), or β-arrestin2 in combination with siCXR7 (βAR2siR7) and stimulated with EGF (+). The western blot experiments were repeated twice and one representative experiment is shown. **E-H**. Following transfection with EGFR-WT plasmid for EGFR over expression (O.E.), PLA was performed in MCF7 cells to visualize heterodimerization of CXCR7 with EGFR after 5 min EGF (10 ng/mL) stimulation; nuclei were counterstained with DAPI (blue). **E** Incubation with non-specific isotype antibody (negative control). **F**. PLA signal indicating CXCR7/EGFR dimers in non-stimulated MCF-7 cells. **G**. PLA signal indicating CXCR7/EGFR dimers in MCF7 cells transfected with EGFR-WT plasmid and siRNA against βarrestin2. **H**. CXCR7/EGFR dimers in MCF7 cells transfected with EGFR-WT plasmid and siRNA against CXCR7 (630x).

To assess whether down regulation of CXCR7 affected the mitogenic pathway, we assessed for changes in the MAPK/ERK kinase cascades. Since ERK1/2 is classically involved in cell proliferation and differentiation of many cell types, and is the last step in this cascade of events, we analyzed for its expression. The inhibition of CXCR7 by siRNA resulted in decreased ERK1/2 phosphorylation in EGF (10 ng/mL, 2 min) stimulated MCF7 cells (Figure 5A). We also stimulated with SDF-1a, but were not able to observe ERK1/2 activation (data not shown). Since EGFR is a known mitogenic driver of cell proliferation and we demonstrated co-localization between CXCR7 and EGFR earlier, we next analyzed whether EGFR activation was also affected by CXCR7 depletion. Down regulation of CXCR7 decreased activation of EGFR at Tyrosine_1110_ compared to control siRNA transfected cells; quantification of EGFR_Y1110_/EGFR total ratio for EGF stimulated cells in bar graphs underneath blots (Figure 5C). Furthermore, we confirmed these results in EGFR over expressing cells. CXCR7 depletion (siCXCR7) decreased phosphorylation at the Tyrosine_1110_ site in EGFR of over expressing-MCF7 cells, compared to cells over expressing EGFR alone, demonstrating a significant role of CXCR7 in EGFR activation (Additional file 2: Figure S2).

### β-arrestin2 plays a role in CXCR7 mediated phosphorylation of EGFR and ERK

CXCR7 is known to signal through β-arrestin2 in a variety of cell types including rat vascular smooth muscle cells, mouse medial ganglionic eminence interneurons, HEK cells, and normal human epidermal melanocytes [8, 24–26]. To evaluate the role of the CXCR7/β-AR2 axis in the EGFR mediated mitogenic pathway, we over expressed β-AR2 in MCF7 cells, which express a relatively low basal level of β-AR2. We over expressed β-AR2 in the presence or absence of CXCR7 siRNA and analyzed phosphorylation levels of ERK and EGFR. Our studies show increased phosphorylation of ERK1/2 and EGFR at Y1110 in β-AR2 over-expressing cells. However, with CXCR7 down regulation, we observed a significant decrease in phosphorylation of both ERK1/2 and EGFR_Y1110_ even after β-AR2 over expression (Figure 5B,D). In order to visualize whether β-AR2 is involved in the colocalization of CXCR7-EGFR, we used siRNA to down regulate β-AR2, followed by EGF stimulation and PLA to assess the level of CXCR7-EGFR heterodimerization. As MCF7 cells express relatively low levels of EGFR, we concomitantly employed a pLPCX-vector construct to over express EGFR. Non-specific isotype control antibodies were used as negative control (Figure 5E). EGFR over expressing cells showed a dramatic increase of CXCR7-EGFR co-localization compared to non-transfected cells (Figure 5F). However, siRNA mediated knockdown of β-AR2 in EGFR overexpressing MCF7 cells resulted in an almost complete loss of CXCR7-EGFR colocalization events (Figure 5G). Similarly, down regulation of CXCR7 via siRNA as a control, significantly diminished CXCR7-EGFR colocalization (Figure 5H).

## Discussion

### CXCR7 is expressed across breast cancer cell lines

We observed that cell lines with higher expression of CXCR7 are also estrogen receptor positive (ER+) and of luminal cell origin. We also observed that CXCR7 mRNA expression was distinct from CXCR4 mRNA expression when normalized to a house-keeping gene PPIA. Protein expression does not always correlate with mRNA level, therefore we cannot assume that the protein level of CXCR7 is also higher than that of CXCR4 in the same cell line when comparing the intensity of specific bands in immunoblots as band intensity is dependent on the affinities of the antibody used for probing the protein, e.g., CXCR4 vs CXCR7. In tissues, CXCR7 expression was mostly restricted to the luminal layer of breast ducts in normal breast tissue and was highly over expressed in tumor tissue. Boudot et al. showed that the CXCR7 axis is under the control of the estrogen receptor and that CXCR7 expression is decreased upon addition of estrogen to MCF7 cells [22]. It is important to consider that many of the breast tumors that metastasize to bone are ER + [27]. Interestingly, we consistently found CXCR7 over expression predominantly in ER + cells, thus exploring CXCR7’s role in metastasis independently of CXCR4 is highly relevant [28]. Moreover, EGFR expression correlated with CXCR7 expression across breast cancer cell lines and no EGFR expression was observed in the cell lines that lack CXCR7 protein expression. This may support the role of the heterotypic and unique CXCR7-EGFR interaction in breast cancer proliferation.

### Down regulation of CXCR7 decreases growth of breast cancer cells

Clonogenic assays showed a significantly slowed reproductive potential of CXCR7 depleted MCF7 cells compared to control cells (Figure 2A, inset). We confirmed the role of CXCR7 in proliferation in different breast cancer cell lines and found significantly decreased proliferation following CXCR7 down regulation, whereas CXCR7 over expression resulted in an increased proliferation. Consistently, the role of CXCR7 in proliferation has been previously described for other tumor entities, such as prostate cancer [13]. Reduced expression of CXCR7 coincided with an inhibition of cell cycle progression and marked changes in key proteins involved in cell cycle regulation. Cell cycle arrest at the G2/M checkpoint is characterized by increased p21 expression which prevents formation of the Cyclin B/Cdk1 complex necessary for cells to go through mitosis [29]. We observed that CXCR7 depletion increased p21 and decreased Cyclin B1 accumulation, thus preventing the tumor cells from dividing. Consistently, Singh and Lokeshwar showed that down regulation of CXCR7 in prostate cancer cells resulted in similar reduced Cyclin B1 and increased p21 expression [15].

### CXCR7 affects BrCa proliferation in a ligand-independent fashion

MCF7 cells endogenously express the ligand for CXCR7, SDF-1a [30, 31]. Interestingly, this endogenous ligand production is not substantial enough to trigger autocrine downstream events [1]. However, the ligand-independent role of CXCR7 has not been explored in breast cancer. Our results demonstrate that SDF-1a did not increase MCF7 cell proliferation, supporting the conclusion that the effect of CXCR7 in these BrCa cells is ligand independent. However, in vivo, CXCL12 ligand may be expressed at higher levels than those observed in cell cultures [32, 33] and small molecular competitive inhibitors of CXCR7, such as antagonists of SDF-1a binding have been shown to decrease tumor growth in lymphoma and lung carcinoma [1]. We also addressed the possibility of a non-canonical ligand of CXCR7, MIF, having an effect on proliferation. MIF is likely a stromal component of the tumor microenvironment, therefore, it is possible that a MIF inhibitor did not have specific cytotoxicity but non-specific cytotoxicity. However, similar to the effects of SDF-1a, it is likely that *in vivo*, stromal components may change the behavior of CXCR7 significantly. Furthermore, we conducted proliferation studies with and without EGF treatment in cells with control (shC) and low (shR7) CXCR7 levels to assess for the proliferative effects of EGF on our postulated CXCR7-EGFR interaction. We confirmed that following CXCR7 depletion in MCF7 cells with ~50% stable CXCR7 down regulation, proliferative stimulation of EGF was limited.

### CXCR7 affects BrCa proliferation in a CXCR4 independent fashion

Previous studies have shown that elevated expression and function of CXCR4 have significant effects on survival, proliferation and metastasis of BrCa [34, 35]. CXCR7 has also been directly linked to metastasis through its interaction with CXCR4 [1, 13, 28, 36, 37]. In some cells it has been shown that CXCR7 and CXCR4 cooperate to mediate signaling and we aimed to investigate whether this is the case in breast cancer cell lines [26, 38]. Our results demonstrate that down regulation of the CXCR7 receptor resulted in a significant decrease in proliferation. In contrast, decreased expression of CXCR4 appears to increase proliferation to levels near those observed by CXCR7 over-expression, however, the effects were not statistically significant (Figure 2C). CXCR7 and CXCR4 levels are closely regulated across cell lines and CXCR7 is known to affect the stability of CXCR4 [39], therefore it is likely that expression levels of one receptor affect the other. However, since our results demonstrate that solely CXCR7 expression had a consistently significant effect on proliferation, we can infer the distinctive and co-receptor independent effect of CXCR7 in MCF7 cell proliferation.

### CXCR7 interacts with EGFR in human breast cancer cell lines and tissues

Several reports have linked CXCR7 with other marker proteins including CD31 and EGFR [15, 32, 37]. Our results demonstrate for the first time that CXCR7 interacts with EGFR in human breast cancer cell lines and that CXCR7-EGFR co-localization is significantly increased upon EGFR over expression. To corroborate our results and evaluate its clinical significance, we analyzed human breast samples for colocalization of CXCR7 and EGFR. We observed a significant increase in receptor colocalization in human ER + breast cancer tissues compared to normal breast tissues suggesting an important role of the CXCR7-EGFR association in ER + breast tumor cell proliferation.

### CXCR7 interacts with CD31 in human breast cancer tissues

Furthermore, CXCR7 is known to be expressed in endothelial cells and tumor associated vasculature [13, 40]. Consistently, we show that CXCR7 co-localizes with the endothelial cell marker CD31 in breast tumor vessels. This is also in accordance with analysis of the Oncomine database which identifies CXCR7 as over expressed in breast tumor associated stroma when compared to normal tissue stroma (Additional file 1: Figure S1). Expression of CXCR7 in the tumor associated vasculature and stroma suggests a role in endothelial cell proliferation and underlines the importance of CXCR7 for tumor growth and microenvironment.

### Down regulation of CXCR7 decreases ERK1/2 and EGFR activation

Seven transmembrane receptors have been shown to heterodimerize with ERBB family members and studies have demonstrated that the CXCR7 co-receptor CXCR4 interacts with EGFR in other types of cancer, like tumors of the prostate and bladder [37, 41]. EGFR is also known to be transactivated by seven transmembrane receptors to regulate events such as ERK signaling and proliferation [10]. We found that down regulation of CXCR7 decreased the level of activation of phospho-EGFR and phosho-ERK1/2, which suggests a strong association of CXCR7 with EGFR in breast cancer in accordance with our in situ co-IP data and confirms a role of CXCR7 in EGFR mediated ERK signaling. Additionally, our studies using RNA interference indicate that disruption of the CXCR7-EGFR interaction following down regulation of CXCR7 not only affects the phosphorylation activity of EGFR and ERK1/2 but leads to cell cycle arrest and decreased cell proliferation.

### CXCR7 affects phosphorylation of EGFR and ERK via β-Arrestin2

Following binding of SDF-1a, CXCR7 preferentially induces signaling events via interaction with β-AR2 [8]. β-arrestins can interact directly with component kinases of the extracellular signal regulated kinase ERK/MAPK cascades leading to metastable β-arrestin complexes with ERK1/2 [9, 42]. β-arrestins are also relevant in breast cancer progression, particularly β-AR2 which is expressed across both, luminal and basal breast cell lines [43]. However, it was not clear yet whether β-arrestins also mediate ligand independent CXCR7 signaling. Therefore it was necessary to determine whether CXCR7 affects the mitogenic pathway via interaction with EGFR directly or indirectly through β-AR2. Our phosphorylation studies show an increased phosphorylation of ERK1/2 and EGFR at Y1110 in β-AR2 over expressing cells. Importantly, CXCR7 down regulation resulted in a significant decrease of phospho ERK1/2 and EGFR _Y1110_ even after β-AR2 over expression. Furthermore, we found that decreasing β-AR2 expression substantially decreased the colocalization of CXCR7-EGFR and β-AR2 thus plays a significant role in their association. These results suggest that the CXCR7/β-AR2 axis plays a key role in activation of EGFR and ERK and the CXCR7-EGFR crosstalk to mediate breast tumor proliferation.

## Conclusions

**Figure 6:**
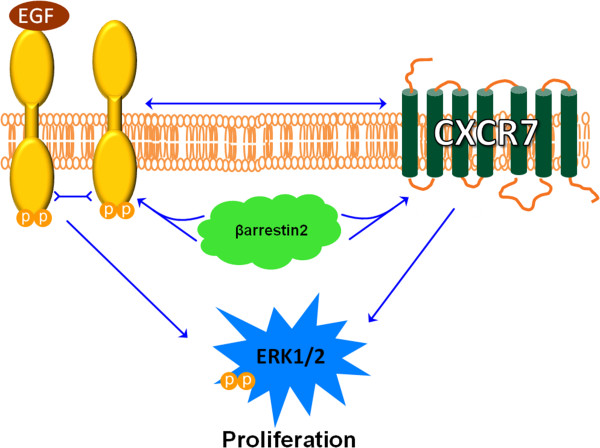
**A model of CXCR7-EGFR Interaction.** CXCR7 signals through β-arrestin2, which mediates interaction of CXCR7 with EGFR and activation of EGFR mediated signaling. In some instances CXCR7 might activate mitogenic signaling independently from EGFR (and vice versa). This EGFR transactivation, which occurs in a CXCR7-ligand independent fashion, induces proliferation effects in breast cancer cells.

In the present work we demonstrate that CXCR7 is an important modulator of cell proliferation through regulation of cell cycle progression and mitogenic activation of CXCR7-expressing BrCa cells. Interestingly, we found a ligand independent role of CXCR7 mediated cell proliferation.Our data demonstrate a significant heterotypic molecular interaction between two cell surface receptors with relevant clinical significance. We investigated the interaction of CXCR7 with EGFR and its role in BrCa cell proliferation and progression. We demonstrate that CXCR7 employs ERK1/2 mediated proliferation via the scaffold protein β-AR2 as its aid to interact with EGFR and offer a model for this interaction (Figure 6). New therapeutic strategies should employ relevant CXCR7 C-terminus inhibitors, not just the currently available ligand-like inhibitors to disrupt the CXCR7 mediated cancer phenotype. Therefore, exploring the role of CXCR7 in BrCa progression is clinically relevant and targeting the CXCR7-EGFR interaction might be a first line of therapy for patients presenting with primary tumors with CXCR7 over expression.

## Materials and methods

### Cell lines and cell culture

All cell lines were obtained from an authenticated source (ATCC, Manassas, VA) and used within six months of resuscitation of original cultures. The cell lines used in the study were also authenticated for their origin by Genetica DNA Laboratories Inc (Cincinnati, OH). Culture and maintenance of BrCa cell lines were performed by routine cell culture procedures. Cells were cultured in RPMI 1640 medium supplemented with fetal bovine serum (FBS, 10%) and gentamicin (10 μg/ml).The normal breast epithelial cell line, MCF10A was maintained in MEBM medium supplemented with the additives obtained from Lonza/Clonetics Corporation as a kit: MEGM Kit Catalog No. CC-3150. The normal breast epithelial cell line, MCF12A was maintained in DMEM/F12 medium supplemented with the additives obtained from Lonza/Clonetics Corporation as a kit: MEGM Kit Catalog No. CC-3150 and 10% horse serum.

### Gene knockdown with siRNA

Cells cultured for 24 h were transfected with gene-specific 21-mer siRNA sets (Smartpool siRNA, Dharmacon/Thermo Scientific Inc, Chicago, IL), using the Dharmafect-2 transfection protocol.

### Stable silencing of CXCR7 expression by shRNA

MCF7 shCXCR7 and vector control cell lines were made as described before [44]. Briefly, CXCR7 shRNA and scrambled sequence shRNA (Control shRNA) constructs were cloned into a pRS plasmid under the control of a U6 promoter for stable expression (HuSh-29mer, Origene Technology Inc. Baltimore, MD). Cells were transfected with shRNA-pRS using Lipofectamine 2000 (In Vitrogen Inc., Carlsbad, CA). Stable transfectants were selected from transfected cultures following two weeks of culture in puromycin selection medium (2.0 μg/ml). Emergent cell colonies were evaluated for CXCR7 mRNA knockdown by q-PCR and immunoblotting.

### Gene over-expression with cDNA

#### Cells were transfected with a human full-length CXCR7

cDNA (pCMV6-Neo vector; OriGene, Rockville, MD) or CXCR7-green fluorescent protein (EGFP-N1 vector; BD Biosciences, San Jose, CA), a generous gift from Dr. Katherine Luker University of Michigan, Ann Arbor MI [45]. EGFP-β-arrestin2 cDNA was a generous gift from Dr. V.R. Krishna Jala (Department of Immunology, University of Louisville, Louisville, KY). EGFR insert was cloned into a pLPCX plasmid under control of a CMV-IE promoter (Clontech, Mountainview, CA). Cells were transfected using Lipofectamine 2000 and analyzed for over expression by q-PCR and immunoblotting.

#### Cell proliferation assay and cell cycle phase analysis

Cell proliferation and cell viability were determined by cell counting and MTT reduction assays, respectively. Cell cycle phase-fractionation was conducted using preparations of propidium iodide labeled nuclear suspension analyzed on a Beckman-Coulter XCEL flow cytometer as described before [46]. The MIF inhibitor used for cell proliferation was 4-IPP (Tocris Bioscience Cat. No. 3429).

### Quantitative real time PCR (q-PCR)

Total RNA isolated from cells 48 h later was subjected to cDNA synthesis and q-PCR using iQ SYBR-Green Supermix (Bio-Rad, Hercules, CA) and the primers described in Additional file 3: Table S1 (Supplement). The mRNA levels were normalized to that of the housekeeping protein, peptidylprolyl isomerase A (PPIA) based on the threshold cycle (C_t_) of each sample in RT q-PCR. Relative levels of mRNA expression were calculated from ΔC_t_ where ΔC_t_ = (test mRNA Ct- PPIA Ct). Values are shown as fold difference (FD), defined as FD = (2^ΔCt^)^-1^ × 100).

### Immunoblotting

Expression of specific proteins in treated cells was analyzed by routine immune-blotting technique. Briefly, proteins were quantified using Micro BCA^TM^ Protein Assay Kit (# 23235, Thermo Scientific, Lafayette, CO) and equal amounts of proteins were loaded and separated on 10% SDS acrylamide gels and blotted onto PVDF Immobilon-P Membrane (EMD Millipore, Billerica, MA). Presence of specific proteins on the membranes was detected using specific antibodies and the ECL + Kit (GE Health Science). Relative protein band intensities were quantified using densitometry (Gel Logic 2200, Care-Stream Instruments, Rochester, NY). Band densities were normalized to those of actin.

### Antibodies

Antibodies used as described in [15] for cell cycle analysis and isotype controls; in addition, specific antibodies used are: CXCR7 Ab (GTX1000027, GeneTex, Irvine, CA) for confocal at 1:500; CXCR7 Ab (20423-1AP, Proteintech Group, Chicago, IL) 1:5000 for immunoblotting; EGFR Ab (E2760, Sigma Aldrich, St. Louis, MO) for confocal 1:500; EGFR Ab (Cell Signaling 2646S, Beverly, MA) for western blotting at 1:3000; phospho-EGFR_Y1110_ Ab (2284–1, Epitomics, Cambridge, MA) for western blotting 1:1000; β-arrestin2 Ab (sc-13140, Santa Cruz, Dallas, TX) for western blotting 1:500.

### Tissue specimens

Resected human breast tissues were obtained with patient’s informed consent and were fixed in formalin, paraffin embedded and sectioned under an approved Institutional Review Board protocol at the University of Miami, FL. All specimens used in this study were blinded to protect patient identities as per HIPPA and IRB regulations.

### Proximity ligation assay

Proximity Ligation Assay (PLA), also called in-cell co-IP assay, by Olink Bioscience (Sweden), was used to identify specific colocalization events of CXCR7 and EGFR in fixed cells or tissues. Prior to the PLA, cells were fixed in 4% paraformaldehyde at 4°C, 15 minutes and human breast tissues were de-waxed. Cells and tissue were incubated with anti-CXCR7 rabbit IgG (1:500, GeneTex) and anti-EGFR mouse monoclonal antibody (1:500, Sigma) or normal rabbit IgG. Following incubation with primary antibody, cells were incubated with corresponding secondary antibodies that were conjugated with oligonucleotides (PLA probe MINUS and PLA probe PLUS). Then, ligation solution was added, consisting of ligase and two oligonucleotides that hybridize to the two PLA probes and form a closed circle if the PLA probes are in close enough proximity. Strand extension by rolling circle amplification (RCA) by T4-ligase and PCR amplification of double hybridized DNA was performed as described by the supplier. Fluorescently labeled oligonucleotides were used detection of the RCA product. The resulting signal (a red fluorescent signal of Texas-Red fluorescent tagged amplified DNA) occurs wherever the two molecules are colocalized. Quantification of the confocal images was performed using the Duolink Image Tool software (Olink Bioscience).

### Image acquisition and analyses

Cells and tissues were viewed under a Zeiss LSM 700 laser scanning confocal microscope. Images were acquired at 400× to 630× magnification using 40× or 63× oil-immersion objectives. Hardware control and acquisition of images was performed using the ZEN software.

### Oncomine box plots

CXCR7 expression analysis performed using Oncomine, a cancer microarray database and web-based data-mining database platform [47]. We used subsequent databases based on mRNA expression collected via Affymetrix X3P Array. Filters were applied for differential analysis comparing specific classifications of breast cancer to normal in breast tissues. Box plots represent CXCR7 expression changes in the Ma Breast 4 database as measured by Affymetrix Human X3P Array in Ma Breast 4 database.

### Statistical analysis

All quantitative data shown, except the western blot quantification, were from three separate experiments each data point representing a mean of triplicate determination. Western blots have been repeated twice. Significance of data was analyzed with the Prism graph pad software. Immunohistochemistry data were independently evaluated by two investigators.

## Electronic supplementary material

Additional file 1: Figure S1: CXCR7 co-localizes with CD-31 in breast tumor associated stroma. Expression of CXCR7 and endothelial cell marker CD31 in human breast tissue was analyzed by immunofluorescence. A. Nuclei of human breast tissue specimen counter stained with DAPI B. CXCR7 (green fluorescence) was mostly restricted to the luminal layer of breast ducts. C. CD31 (red fluorescence) marks endothelium tissue D. Overlay of B and C: CXCR7 (green fluorescence) colocalized with endothelial cell marker CD31 (red fluorescence) within the ER + breast cancer tissue. E-F. Box plots of the Ma Breast 4 Study showing CXCR7 relative mRNA expression as measured by Affymetrix Human X3P Array in Ma Breast 4 database comparing CXCR7 E. In ductal breast carcinoma in situ stroma vs. normal tissue CXCR7 has an Over-expression Gene Rank: 59 (in top 1%), P-value: 1.10E-6, t-Test: 6.304 and Fold Change: 4.431 F. For invasive ductal breast carcinoma stroma vs. normal tissue CXCR7 has an Over-expression Gene Rank of 125 (in top 1%), P-value: 6.08E-5, t-Test: 5.148 and Fold Change: 4.610. (TIFF 17 MB)

Additional file 2: Figure S2: pY1110/EGFR ratio is decreased in MCF7 cells overexpressing EGFR-WT. MCF7 cells were transiently transfected with EGFR plasmid or with both EGFR plasmid and siRNA targeting CXCR7. CXCR7 depletion (siCXCR7) decreased phosphorylation at the Y1110 site in EGFR over expressing MCF7 cells after EGF (10 ng/mL, 2 min). (TIFF 6 MB)

Additional file 3: Table S1: Primer sequences used for analysis of mRNA expression for CXCR7, CXCR4, and PPIA. (DOCX 11 KB)

## Abbreviations

BrCa: Breast cancer
CXCR: CXC-chemokine receptor
EGFR: Epidermal growth factor receptor
CXCL: Chemokine receptor ligand
SDF-1a: Stromal derived factor alpha
ITAC: Interferon inducible T-cell alpha chemoattractant
CD31: Cluster of differentiation 31
EGF: Epidermal growth factor
ER: Estrogen receptor
PLA: Proximity ligation assay
β-AR2: Beta-arrestin-2
PPIA: Peptidylprolyl isomerase A
FD: Fold difference
qPCR: Real time quantitative polymerase chain reaction.
