# Supplementary figures and images for: The chemokine receptor CXCR7 interacts with EGFR to promote breast cancer cell proliferation
Source: Mol Cancer. 2014 Aug 28;13:198. doi: 10.1186/1476-4598-13-198 (PMC4167278; doi:10.1186/1476-4598-13-198)

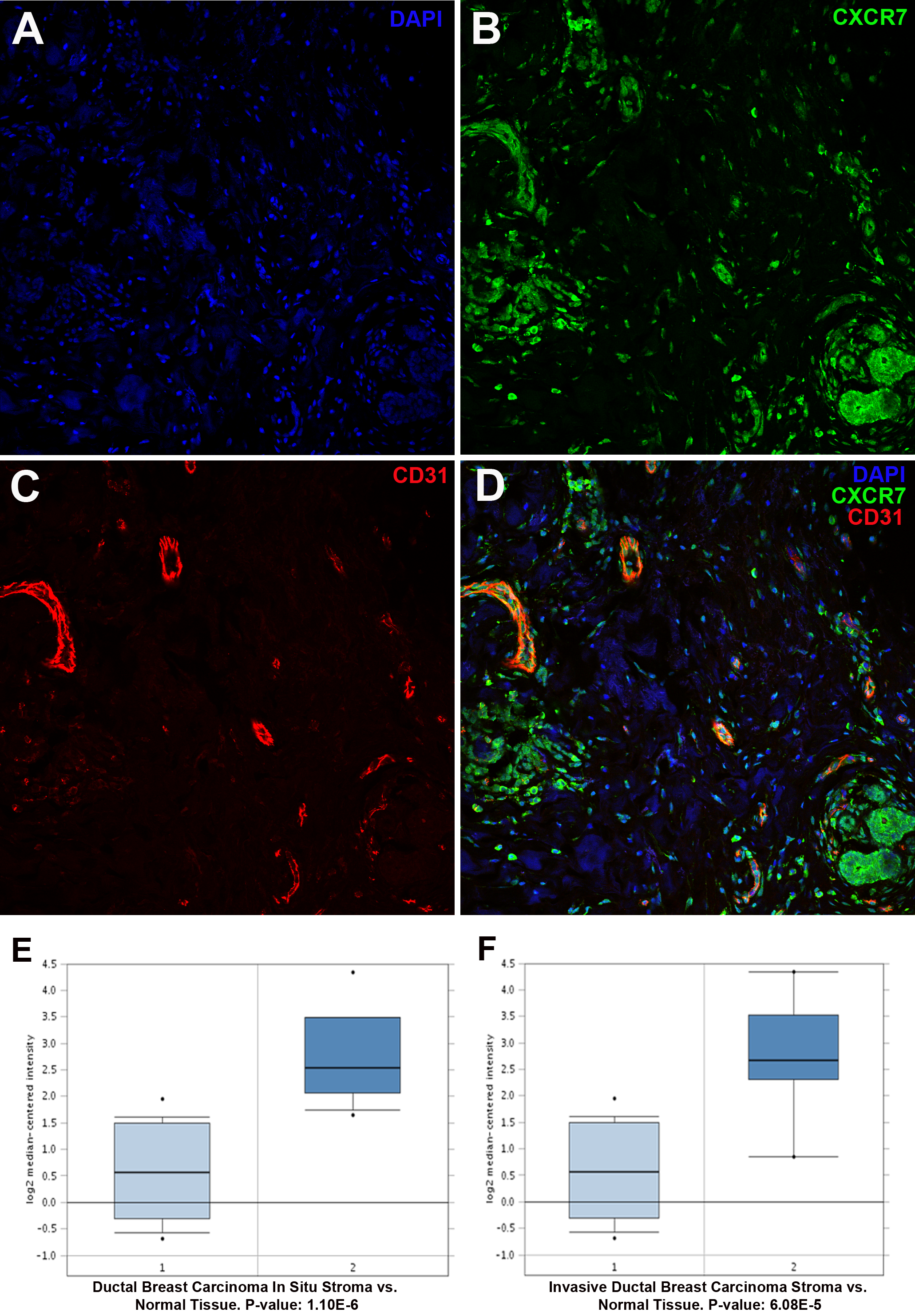

Supplement: Supplementary file 1 — Additional file 1: Figure S1: CXCR7 co-localizes with CD-31 in breast tumor associated stroma. Expression of CXCR7 and endothelial cell marker CD31 in human breast tissue was analyzed by immunofluorescence. A. Nuclei of human breast tissue specimen counter stained with DAPI B. CXCR7 (green fluorescence) was mostly restricted to the luminal layer of breast ducts. C. CD31 (red fluorescence) marks endothelium tissue D. Overlay of B and C: CXCR7 (green fluorescence) colocalized with endothelial cell marker CD31 (red fluorescence) within the ER + breast cancer tissue. E-F. Box plots of the Ma Breast 4 Study showing CXCR7 relative mRNA expression as measured by Affymetrix Human X3P Array in Ma Breast 4 database comparing CXCR7 E. In ductal breast carcinoma in situ stroma vs. normal tissue CXCR7 has an Over-expression Gene Rank: 59 (in top 1%), P-value: 1.10E-6, t-Test: 6.304 and Fold Change: 4.431 F. For invasive ductal breast carcinoma stroma vs. normal tissue CXCR7 has an Over-expression Gene Rank of 125 (in top 1%), P-value: 6.08E-5, t-Test: 5.148 and Fold Change: 4.610. (TIFF 17 MB) [file 12943_2014_1404_MOESM1_ESM.tiff]
