# Supplementary material for: The chemokine receptor CXCR7 interacts with EGFR to promote breast cancer cell proliferation
Source: Mol Cancer. 2014 Aug 28;13:198. doi: 10.1186/1476-4598-13-198 (PMC4167278; doi:10.1186/1476-4598-13-198)
Supplement: Supplementary file 3 — Additional file 3: Table S1: Primer sequences used for analysis of mRNA expression for CXCR7, CXCR4, and PPIA. (DOCX 11 KB) [file 12943_2014_1404_MOESM3_ESM.docx]

| **Additional Table S1**. Primer information | | |
| --- | --- | --- |
| mRNA target | Forward Primer 5'-->3' | Reverse Primer 5'-->3' |
| PPIA | TCATCTGCACTGCCAAGACTG | CATGCCTTCTTTCACTTTGCC |
| CXCR7 | ACAGGCTATGACACGCACTG | ACGAGACTGACCACCCAGAC |
| CXCR4 | TCATCAAGCAAGGGTGTGAG | GGCTCCAAGGAAAGCATAGA |
| **Supplementary Table 1: Primer Sequences used for quantitative real time PCR and procedure** Using the CFX96 Thermocycler, the procedure programmed for all primers are as followed: 95°C for 30 seconds, 40 cycles of 95°C for 1 sec, and 60°C for 25 seconds. To generate a melt curve, a dissociation procedure was performed following the 40 cycles from 55C to 95C at 0.5C increments, 10 seconds each. | | |
